# Online Problem-Based Learning Intervention on Self-Directed Learning and Problem-Solving through Group Work: A Waitlist Controlled Trial

**DOI:** 10.3390/ijerph19020720

**Published:** 2022-01-10

**Authors:** Florence M. F. Wong, Crystal W. Y. Kan

**Affiliations:** 1 School of Nursing, Tung Wah College, Hong Kong, China; 2 School of Health Sciences, Caritas Institute of Higher Education, Hong Kong, China; ckan@cihe.edu.hk

**Keywords:** self-directed learning, problem-solving, problem-based learning, online intervention, waitlist-control trial, nursing

## Abstract

Background: Small group work embraces independent study and interactive learning, which enhance knowledge acquisition and skills. Self-directed learning (SDL) and problem-solving (PS) are essential skills in the development of the nursing profession. During the coronavirus pandemic, virtual learning was indispensable. However, little is known about how students develop SDL and PS abilities through online learning through group work. Objective: To evaluate the effects of the online intervention on SDL and PS abilities through interactive group work. Methods: A randomised waitlist-control trial was carried out. A structured intervention using problem-based learning (PBL) as a guideline was used to direct student learning in small group work. Assessments were scheduled at Time 0 (baseline), Time 1 (8th week), Time 2 (16th week), and Time 3 (28th week). Results: The mean student age was 21.45 (SD = 0.86). About 78% of students were female. There was no significant difference in demographic characteristics and analysis at the baseline. Students in the intervention group reported greater improvement in the SDLRS and PSI at the 8th week, whereas those in the waitlist control group reported greater improvement in the SDLRS and PSI at the 16th week. Sustained effects in the SDLRS and PSI were observed in both the intervention and waitlist control groups at the 16th and 28th weeks, respectively. A repeated-measure analysis was performed to compare the SDLRS and PSI in different periods and revealed statistically significant results (*p* < 0.001) in all subscales of SDLRS and PSI in the four study periods. Conclusions: The guidelines appear to be an effective treatment for SDL and PS ability enhancement with sustainable effects through interactive group work. The guidelines with explicit instructions and learning objectives provide directions and guidance to students to learn more effectively. The educator plays a vital role in facilitating the students’ SDL and PS ability improvement.

## 1. Introduction

### 1.1. Background

Small group work is a well-known teaching and learning pedagogy in nursing education, as it encourages a student-centred learning approach and broadens student learning [1,2,3,4]. It embraces learning and development through independent learning and group collaboration [3,4,5,6]. Effective small group learning based on this premise should include students’ active engagement in learning and a sense of collaboration [3,4,5,6]. Since nursing practice emphasises evidence-based knowledge and skills to ensure optimal patient care, self-directed learning (SDL) has been encouraged to be developed for lifelong learning. In the advanced changes in this complex and sophisticated healthcare service, nurses are required to make immediate and appropriate clinical decisions to promote patient care recovery. During nursing education, students can achieve knowledge acquisition and skill development in the classroom and laboratories. The problem-based learning (PBL) approach has been entrenched to integrate students’ individual learned knowledge and skills during their learning process for a higher quality of education and more competent care for patients [7]. To maintain the quality of clinical care and to reach optimal patient outcomes, PBL is used to develop students’ problem-solving (PS) ability. With the growing utilization of interactive small group learning and PBL methods, it is imperative to better understand how students develop their SDL and PS abilities through preparing themselves to be immersed in a collaborative working environment [8,9]. The results of this study suggest that the guideline can help to increase nurse educators’ awareness to facilitate students’ SDL and PS skill learning through an effective interactive small group learning approach.

### 1.2. Background Literature

Small group work is a well-known teaching-learning pedagogy in nursing education to enhance students’ learning through active involvement in individual study and group interactions [1,2,3,4,6]. This teaching-learning approach embraces advantages in developing both in-depth theoretical knowledge and essential skills [10,11] and provides a good platform for students to assess, formulate, and analyse the problem, generating and consolidating the knowledge and solution together [4,12]. Regarding group interactions in small group learning, students share and broaden their learning through discussion, feedback, and clarification to achieve common goals [1,5,6]. Students also develop self and group values and gain peer support to articulate their thoughts and formulate their viewpoints to cultivate independent learning and problem-solving (PS) abilities and skills [9,13]. Moreover, this approach helps students to create and engage in a learning environment to enhance individual and group developments, such as self-directed learning, team interactions, and skill development [11,14,15].

Self-directed learning (SDL) is a learning process employed to allow learners to obtain self-mastery and ownership of learning to direct, regulate, and be accountable for their learning [16]. Moreover, SDL motivates students to understand their values, interests, weaknesses, and strengths [9,17] and encourages students to promote self-discipline with more accountability and learning motivation [18,19]. They can use their acquired knowledge and skills for better decision-making, enhancing competence and confidence [17,20]. With the advantages of the current advanced digital technology, student SDL attitudes can be promoted to foster personal and professional development [6,21,22].

Not limited to knowledge enhancement, evidence documented that core higher-intellectual skills, such as communication, critical thinking, leadership, problem-solving, management, and collaborative skills, can be developed through interactive small group learning [5,10]. In the last few decades, technology has rapidly improved in healthcare settings. Such advanced and sophisticated technology has promoted patient recovery and healthcare services on the one hand, and on the other, nurses acquire a great deal of accountability to make immediate and appropriate clinical decisions. PS ability is an essential skill that includes the process of identifying a problem, analysing its existence and impacts, and critically considering possible solutions to the problem. Inadequate PS ability can be the cause of students’ failure in performance [7,22,23,24,25]. Today, PS skill training is emphasised, but its assessment is usually neglected in the current nursing education.

A recent systematic review reported that interactive small group work with an appropriate teaching-learning strategy develops students’ competence in both SDL and PS abilities through independent study and group learning [26]. PBL is known as a favourable teaching-learning method. This learning method allows students to recall what they have learned, identify what they want to know, and discover the way they can learn to solve the problems [7,23,27]. PBL provides important benefits in knowledge enhancement and skill development during nursing education [7,9,28,29]. Online teaching has been adopted due to the coronavirus pandemic. This study developed a structured online PBL intervention to enhance SDL and PS abilities in undergraduate nursing students through interactive small group work. The results of this study provide knowledge about the use of the structured online PBL guidelines to enhance students’ SDL and PS abilities for personal and professional development through an interactive small group approach.

### 1.3. Aims

This aim of this study was to examine the effects of the structured PBL intervention via online on SDL and PS abilities in undergraduate nursing students through interactive small group work.

## 2. Materials and Methods

### 2.1. Design

A waitlist-control trial with a randomised design was adopted with a total of 299 students. They were divided into two groups and randomly assigned to take the 7-week clinical practicum in two different periods. The first group of students were assigned to the intervention group. They received the intervention first, and then they had 7 weeks of clinical practice after the completion of the group work. After seven weeks, the second group of students in the waitlist control group received the intervention after their 7 weeks of clinical practice.

### 2.2. Conceptual Framework

**Figure 1:**
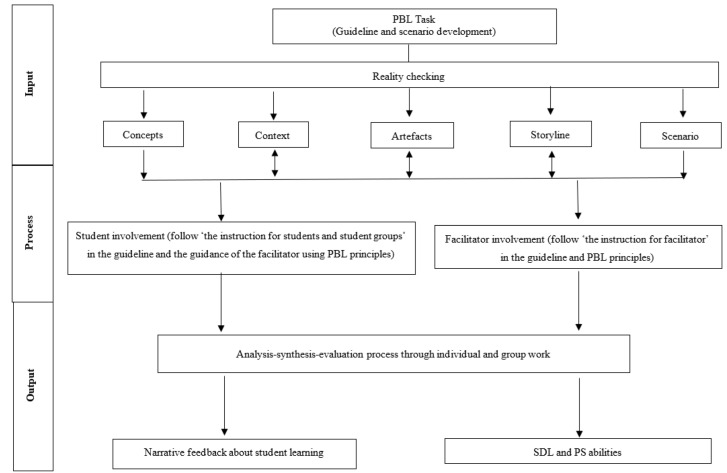
Conceptual framework of input-process-output (IPO) (Albion and Gibson 1998).

The input-process-output (IPO) model developed by Albion and Gibson [30] acted as the conceptual framework in this study. It consists of three dimensions: input, process, and output. The input is a requirement from the environment, including the facilitator and the online structured PBL guideline. The scenario development for PBL tasks consists of concepts, context, artefacts, storyline, and scenario. The context is related to the environmental elements and the problems are identified. The artefacts are the sub-problems that can be produced by a main problem and can be gathered and solved through a step-by-step solution (problem-solving process). The storyline seeks to understand and explain the problem and then describe the problem-solving progress and possible resolutions. The process includes operations and activities that mediate between the inputs and the outcomes. The outputs are the consequences of the process actions. Figure 1 illustrates the conceptual framework of the PBL approach.

### 2.3. Online Structured PBL Guideline

The online structured PBL guideline was designed for small group work based on the IPO conceptual framework and the work by Cadorin et al. [19] and Meo [31]. The guidelines were designed to enhance student SDL and PS abilities through briefing, peer support, free discussion, Socratic questioning, guided reflection, tutor facilitation, and group member and tutor feedback. The educator first formulated a PBL case scenario with an authentic problem. To allow students to learn little by little and step by step, the educator set learning objectives and guiding questions of the fragmented case scenario in each lesson. Supplementary Materials S1 shows an example of case scenario using PBL approach to guide students to think and achieve the respective learning objectives. They were encouraged to establish relevant learning objectives to broaden their learning through self-study and group discussions. Peer–peer interaction through group discussion provided a good platform for students to reflect, give feedback, and evaluate their learning. The educator played important facilitating roles to guide students to think, learn, and work independently and in a group through a variety of resources. The educator encouraged students to perform self-evaluation with adequate support and feedback for individual and collaborative learning. Table 1 illustrates the online structured PBL guidelines for small group learning in detail.

### 2.4. Setting and Participants

Participants who were undergraduate nursing students and had been involved in interactive small group work were included in this study. Eligible students were recruited from the bachelor’s in nursing programme at a professional training institution. They were randomly assigned to either the intervention or the waitlist control group by the computerised system. The priori power analysis was performed to reach the desired power of 0.80 and a Type I error of 0.05 with an effect size of 0.4 using G-Power. The sample size was calculated, and a minimum of 200 students (100 students in each group) were needed.

### 2.5. Tools for Outcome Measures

One set of questionnaires was used to collect demographic characteristics (age, gender, and average hours of small group work), the Self-Directed Learning Readiness Scale (SDLRS), and the Problem-Solving Inventory (PSI).

The SDLRS was developed by Fisher et al. [32] and used to assess student perception of skills and attitudes related to their self-directedness in learning. This tool consists of 40 items with a five-point Likert scale from 1 (strongly disagree) to 5 (strongly agree). The SDLRS includes three subscales: self-management (SM; 13 items), desire for learning (DL; 12 items), and self-control (SC; 15 items). The SM subscale reflects the characteristics of an individual being able to manage his or her learning. The DL subscale is associated with an individual’s desire for learning. The SC subscale is related to the features of self-control and being in control of one’s learning. The ranges of the overall, SM, DL and SC scores were from 40 to 200, 13 to 65, 12 to 60, and 15 to 75, respectively. Higher scores on the SDLRS indicate stronger SDL ability. Mean scores greater than 150 indicate a high level of SDL ability. The internal reliability values for Cronbach’s alpha for the overall SDLRS, SM, DL, and SC in this study were reported as 0.899, 0.872, 0.871, and 0.903, respectively, indicating very good reliability.

Heppner and Petersen [33] developed the PSI that consists of 32 items with a six-point Likert scale ranging from 1 (strongly agree) to 6 (strongly disagree). It is used to measure an individual’s perceptions regarding his or her PS abilities and PS style in daily life. The PSI includes three subscales: approach-avoidance style (AAS; 16 items; score range from 16 to 96), problem-solving confidence (PSC; 11 items; score ranging from 11 to 66), and personal control (PC; 5 items; score ranging from 5 to 30). The PSC effectively assesses self-perceived confidence, belief and self-assurance in solving problems. The AAS measures an individual’s tendency to approach or avoid problems. Finally, the PC assesses elements of self-control of emotions and behaviour. A higher AAS reflects more avoidance than approaching problems. A higher PSC indicates lower levels of PS confidence. Lastly, a higher PC signifies a more negative perception of personal control of problems. The total score of PSI ranges from 32 to 192. Lower scores on each subscale and the total PSI indicate higher functional PS abilities. The internal reliability values for Cronbach’s alpha of the overall PSI, AAS, PSC, and PC in this study were reported as 0.751, 0.799, 0.772, and 0.649, respectively, indicating good reliability.

Due to the smoothness of the study, English versions of these two instruments with the authors’ permission were adopted. To increase their applicability in this study, two experts in nurse education reviewed and verified them. A pilot study for tool validity was also conducted in 20 undergraduate students who were not included in this study. The results of the internal consistency, with Cronbach’s alphas, of the SLDR and PSI are reported in Table 2.

### 2.6. Data Collection

All eligible students were invited and asked to complete one set of questionnaires before the course started (T0). The first group of students were assigned to the intervention group and were required to form a group of five to seven members. Only one tutor (the principal investigator) acted as the facilitator to provide case information, supervision, guidance and support and monitor individual and group learning. The communications between students and the tutor included regular meetings via Blackboard Collaborate Ultra and other channels, such as Zoom meetings and WhatsApp.

**Figure 2:**
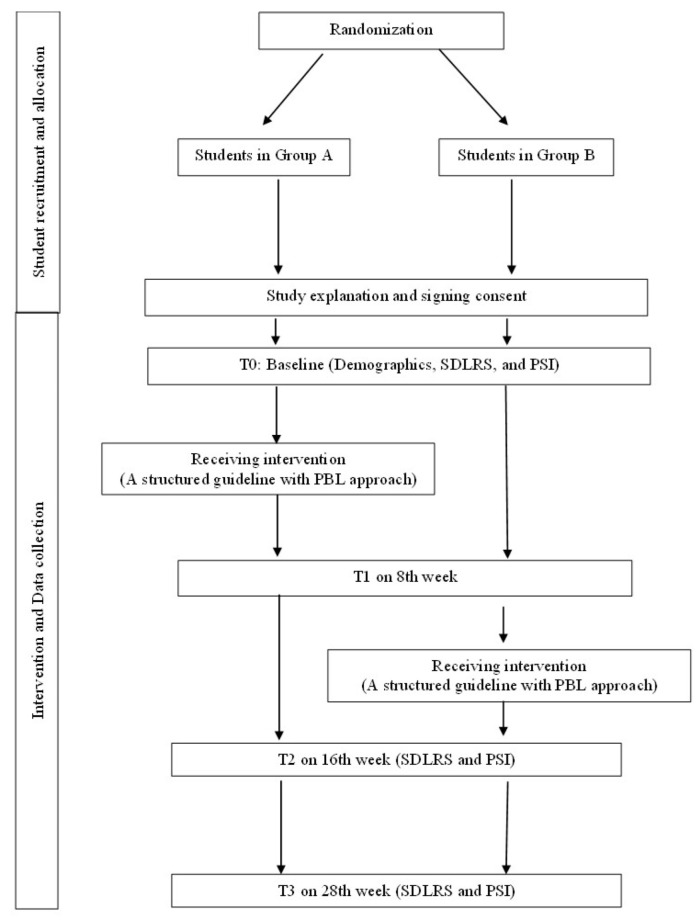
Flow diagram of student recruitment and allocation.

In the first lesson, students received clear instructions with rules and regulations, and their roles and responsibilities were explained concerning working on a small group project. Each group would have an individual case scenario with learning objectives. In each lesson, students were expected to take on individual tasks for self-study and share their learning in the group. Before the next lesson, students were required to submit their learning records to the tutor. During the 8th week, all students in the intervention and waitlist control groups were required to complete the questionnaires (SDLRS and PSI) (T1) again. Students in the waitlist control group started the course and received the same instructions and intervention as the first group on the 8th week. All students were required to complete the questionnaires (SDLRS and PSI) during the 16th week and 28th week. Figure 2 shows the flow diagram of student recruitment and allocation.

### 2.7. Ethical Considerations

Ethics approval was obtained from the study professional training institution. All students were assured that they could withdraw from the study without any accountability at any time. All data related to personal information were kept confidential. Student numbers were recorded to link to the same students in the code list for data collection in the four study periods. The records with the student numbers were destroyed after all data entry was completed.

### 2.8. Data Analysis

Statistical analysis was performed using the IBM SPSS software (v.26) (SPSS Inc., Chicago, IL, USA). Differences in subjects’ characteristics (age, gender, and hours spent in small group work per week and levels of SDLRS and PSI in the four study periods: baseline, 8th, 16th and 28th weeks after intervention) between the intervention and control groups were examined using t-tests (continuous data) and the chi-square test (categorical data).

The intervention effect was evaluated by comparing the intervention and control groups. A repeated-measure analysis of variance (ANOVA) was performed to detect the effects of the outcome measures (SDLRS and PSI) with time (pre-intervention, post-intervention and follow up) as the within-subject variable and the main effects of time and group (intervention and control groups) as the between-subject variable. For all models, the normality of the regression residuals was examined. All statistical tests involved were two-sided, and a *p*-value < 0.05 was considered statistically significant.

## 3. Results

### 3.1. Participant Characteristics

A total of 199 students participated in the study, including 101 (50.8%) from the intervention group and 98 (49.2%) from the waitlist control group. There was no attrition. The mean age was 21.45 ± 0.86 years old (ranging from 20 to 25 years). No significant differences in age and gender were found, but the hours spent on small group work were different between the two groups. Table 3 illustrates the student characteristics in detail.

### 3.2. Analysis of Outcomes

#### 3.2.1. SDLRS and PSI between Two Groups in Four Stages

Table 4 illustrates the descriptive analytical results of the four study periods. The results of this study demonstrate that students in the intervention group performed better on the SDLRS and PSI from the baseline to the 8th week. The scores of all SDLRS subscales were maintained, and the performance on the PSI subscales was continuously improved from the 8th to the 16th week and even in the 28th week. Students in the waitlist control group exhibited poorer performance on the SDLRS and PSI at the baseline and 8th week. However, the scores for all SDLRS subscales increased, and the performance on the PSI subscales improved from the 8th to the 16th week and even in the 28th week.

The independent sample t-test results to compare the intervention and waitlist control groups indicated that all subscales of the SDLRS and PSI were statistically significant at the 8th week. At the 16th week, only the DL and PC subscales exhibited statistically significantly negative differences between the intervention and waitlist control groups. At the 28th week, no significant difference existed between the two groups. Table 5 lists the differences between the SDLRS and PSI of the two groups.

#### 3.2.2. Study Period Changes between Intervention and Waitlist Control Groups

The repeated-measure ANOVA results demonstrate that significant changes occurred in all subscales of the SDLRS and PSI from the baseline to the 8th week (*p* < 0.001). Significant results were also noted in most SDLRS and PSI subscales from the 8th to the 16th week. The sustained effects on SDLRS and PSI were noted, and no significant difference was found from the 16th to the 28th week. Table 4 also illustrates the changes in the subscales of the SDLRS and PSI.

## 4. Discussion

The online structured PBL intervention acting as guidelines appeared to be an effective approach to enhance SDL and PS abilities through interactive small group work in professional education. The conceptual framework was useful in guiding the educator more effectively to organise the tasks and materials for the online PBL [34]. The results of this study suggest that the guidelines made significant improvements in SDL and PS abilities at the 8th and 16th weeks in both the intervention and waitlist control groups. The sustained effects were also observed in the 28th week. The improvement of student SDL and PS abilities was determined by the well-planned intervention with clear guidelines for student learning, the involvement of students and educators, and regular group discussions via various channels. The online structured guidelines with PBL intervention directed and motivated students’ learning initiatives and their self-mastery of learning to deal with problems. The results of this study indicate that the students’ desire for learning and personal control were significantly improved after the intervention. The results of this study also demonstrate the effects of the PBL intervention on SDL and PS abilities, even via online methods. These guidelines provided a comprehensive plan to allow educators to prepare their teaching well to lead and monitor student learning, contributing to students’ knowledge enhancement and skill development.

SDL is an essential component of initiative learning and lifelong learning to motivate students to acquire new information and critically evaluate and apply the information in their studies and future careers [19,22,25,34]. The PS ability enables students to make decisions through a series of critical thinking, logical assessment, and analytical processes [3,29]. As SDL and PS abilities are closely related [23,27,29], the nature of small group work with independent learning and group learning greatly promotes student SDL and PS abilities to achieve learning objectives. This study revealed the short- and long-term benefits of SDL and PS skill enhancement are already reported in this study. The development of two skills is a challenging learning process that requires the involvement of both educators and students. Students with better SDL ability can be highly motivated [23,29,35]. Students enhance their knowledge in a specific area and learn more comprehensively through sharing, discussions, and criticisms, while small group learning embeds self-motivated learning and interactive learning [14,25,36]. Students with SDL and PS skills are more likely to apply knowledge appropriately in different situations to enhance their competence, particularly when dealing with new or complicated clinical situations [7,20,21]. The improvements in student SDL and PS abilities are crucial for increasing their confidence, independence, sense of accountability, and assertiveness for their professional development [15,37]. Educators should evaluate and determine the appropriateness and development of SDL and PS.

PBL is an effective teaching-learning pedagogy for knowledge acquisition and multi-skill development, such as PS, SDL, critical thinking, and clinical reasoning [7,18,23,28,29]. Thus, PBL is crucial to encouraging students to become SDL learners and work more collaboratively [20,23,29]. This study uses online PBL intervention through small group work, confirming improved SDL and PS abilities. A possible explanation for this result is using case segmentation (five parts) to direct students to achieve specific learning outcomes based on the available case information. Students received their focuses of learning for each lesson and searched for new information to achieve their learning objectives. Student interests grew with the learning motivation, promoting better SDL and PS attitudes and behaviours to achieve optimal outcomes [22,26,37]. More importantly, this study also revealed significantly sustained positive effects of the intervention on SDL and PS abilities, which continuously occurred in the two groups after intervention at the 16th and 28th weeks. This study supports the assertion that the utility of the PBL intervention can enhance SDL and PS abilities, which foster lifelong student learning and professional competence. The online PBL intervention through small group work anticipates more focused learning areas, higher learning initiative, and better learning proficiency to achieve more efficient learning outcomes. This learning format facilitates student learning [6,18,36] and provides a platform for students to interact with others so as to develop personal and professional skills through interactive collaboration [6,23,29,38].

Small group work represents a shift from educator-directed teaching to student-directed learning [1,2,3]. Educators play the facilitating role to support and guide students to learn more deeply and effectively through their individual study and peer–peer interaction in a group. As small group teaching and learning pedagogy is commonly adopted in nursing education [24], it facilitates the teaching team and learning environment when students value the interactive activities and learning features that increase their learning satisfaction and knowledge retention [39]. The guidelines adopted in our study include good planning of active learning strategies to accommodate a variety of learning methods using online and PBL modalities. Online learning has been reported for over a decade in previous studies, including in nursing. Maintaining effective interaction among students and with the tutor can be a challenge in small group learning online. This specific teaching-learning method has been examined and has reported significant enhancement in a variety of aspects worldwide, such as self-regulatory abilities [40,41,42]. Online education moves professional education forward to promote student learning and staff development in more effective and quality teaching and learning modalities in different countries [43,44,45]. As today’s technology becomes more advanced, online teaching with student interactions which facilitate student learning with positive outcomes in knowledge acquisition and skill development has become a trend in nursing education [44,45,46].

Regarding the significant positive effects of the PBL intervention during the study periods after the intervention, the educators played an essential role in facilitating student learning following the guidelines. The guidelines employed in this study can give clear directions to the educator(s) and students to enable more interactive teaching and learning engagement. The educators may understand more about individual student learning needs, PS abilities, and group interactive learning and allow autonomous learning to encourage students to engage in self-directed learning activities [19,23]. As SDL is connected to the student’s motivation to learn, the ability to search for resources, and the ability to implement strategies to achieve learning outcomes, educators embrace their vital role in effectively facilitating students to understand their accountability and self-evaluate their learning [16,19]. The educator can provide timely feedback and guidance for students to learn more effectively, progressively improving student SDL and PS abilities [12]. The outcomes of this study encourage educators and programme developers to design structured interventions for quality learning using interactive small groups in nursing education curricula [44,45]. Support is necessary for educators to implement strategies for more positive online learning experiences of students through interactive small groups.

### Limitations

This study is limited to one cohort of Year 4 nursing students from one educational institution. Therefore, the results of this study are difficult to generalise to students in other years or disciplines. Due to the coronavirus pandemic, virtual teaching was adopted, and the online survey was the most significant challenge. Multiple mass and individual emails or other contact methods were used to remind students to ensure responses during the study periods. However, the accuracy of the data may not be guaranteed. As with other studies, although this study reported effective results in improving students’ SDL and PS abilities by small group learning online, knowledge about its effects on SDL and PS in students who resist is inadequate. Further studies to understand the learning of this specific group may be needed.

## 5. Conclusions

Interactive learning through small group activities is favourable for personal and professional development in professional training. This study provides preliminary support for a structured intervention with PBL via an online approach to improve student SDL and PS abilities for both short and long periods. Therefore, the structured guidelines to improve students’ SDL and PS abilities through the individual and group learning format in interactive group work are promising.

## Figures and Tables

**Table 1:** A structured problem-based learning guideline for small group learning.

Aims		
This Guideline Developed Based on Cadorin et al. (2015) and Meo (2013) Provides Clear Instructions for the Tutor and Students to Participate in Small Group Learning.		
Rules and regulations for students	Instruction	Expected outcomes
attain 80% attendance. actively participate in group activities, including group formation, objectives setting, sharing of individual work, discussion, and giving feedback. show and respond with respect and acceptance to others. be accountable for individual tasks and share learning with groupmates. approach tutor anytime when problems encountered by the individual and/or group. submit work on time, including individual tasks to share in the next lesson and the learning reports regarding individual learning and group learning. bear consequences, such as grade deduction, if violating any rules and regulations.	(a) The tutor has to upload students’ rules and regulations to Blackboard before or on the 1st week of the semester. (b) The tutor has to introduce each point of the rules and regulations to students in the 1st lesson. (c) Students have to follow all rules and regulations.	Students are able to ➢ understand their own involvement and accountability of learning. ➢ develop higher intellectual skills, including communication and collaborative skills. ➢ enhance their learning with the support from tutor.
Roles and responsibilities of students		
show a positive attitude and be friendly all the time. show respect and acceptance and be considerate to one another. use positive body language and eye contact. cooperate with other groupmates. be active in setting individual and group learning objectives; individual study and group discussions via various communication channels; and group sharing and discussions to enhance group learning capacity in each meeting. record and submit an individual learning report before each lesson. take turns when giving ideas. approach your tutor when you have problems.	The tutor has to upload students’ roles and responsibilities to Blackboard before or on the 1st week of the semester. The tutor has to introduce each point of the roles and responsibilities to students in the 1st lesson. Students have to follow all roles and responsibilities.	Students are able to ➢ have a clear direction and picture what and how they are involved. ➢ understand their own accountabilities. ➢ develop higher intellectual skills, such as interpersonal communication, problem-solving, and collaborative skills.
Roles and responsibilities of the tutor	Instruction	Expected outcomes
be a facilitator to guide students to meet the learning outcomes. encourage groups to select a leader and assign tasks. monitor and facilitate group discussion. observe and evaluate the involvement of each student in the group. allow students to ask questions, correct misunderstandings and give appropriate advice. reply in simple wording and give easy and appropriate examples. allow groups to showcase their work. evaluate and give feedback on students’ individual and group learning. give groups autonomy and time to prepare high-quality work. always appreciate good attitudes and behaviours as well as goal achievement. conclude the session with concise take-home message.	The course coordinator should introduce the roles and responsibilities to all tutors involved in small group teaching. The course coordinator should explain the logistics and teaching to the involved tutors. All tutors have to meet their students via an online platform at least once a week and provide guidance and support accordingly. All tutors should facilitate and monitor students’ individual learning and in a group. Tutors should support one another if needed.	All tutors have clear direction and understanding to guide their students. may provide their teaching consistently. may enhance their competence in teaching small groups. Students are able to 4. learn more deeply and effectively. 5. have more support and guidance. 6. enhance their learning both individually and in a group.
Roles and responsibilities of a group		
always maintain group dynamics and collaboration in an acceptable manner. set achievable goals and objectives. hold regular meetings and discussions through various channels for group communication. share workload according to individual groupmates’ talents and/or interests. explore the potential of each member to maximise that of the group. make appropriate decisions as a group. strengthen group cohesiveness through effective communication, goal achievement, mutual empowerment, higher job satisfaction, etc.	(a) The tutor has to upload roles and responsibilities of a group to Blackboard before or on the 1st week of the semester. (b) The tutor has to introduce each point of the roles and responsibilities to student groups in the 1st lesson. (c) Student groups have to follow all roles and responsibilities.	All student groups are able to ➢ have a clear direction and picture of what and how they should be involved. ➢ understand their accountabilities. enhance the collaborative dynamics of the group. promote constructive and effective communication. complete their group project more smoothly and efficiently.

**Table 2:** The results of the internal consistence of the SLDR and PSI.

**Internal Consistency** **(Cronbach’s alphas)**	**SM**	**SC**	**DL**	**Overall SDL**	**AAS**	**PSC**	**PC**	**Overall PS**
	0.89	0.91	0.82	0.95	0.90	0.85	0.87	0.95

**Table 3:** Student characteristics (*n* = 199).

	Overall (*n* = 199)		Waitlist-Control (*n* = 98)		Intervention (*n* = 101)		*p*
	*n*	%	*n*	%	*n*	%	
Gender							0.275
Male	43	21.5	18	18.4	25	24.8	
Female	156	78.0	80	81.6	76	75.2	
Age							0.990
Mean (SD)	21.45 (SD 0.86)		21.44 (SD 0.80)		21.47 (SD 0.92)		
20–21	135	67.5	66	67.3	69	68.3	
22–23	55	27.5	28	28.6	27	26.7	
24–25	9	4.5	4	4.1	5	5.0	
Hours spent on small group work (hour)							0.015 *
1	11	5.5	2	2.0	9	8.9	
2	36	18.1	14	14.3	22	21.8	
3	74	37.2	36	36.7	38	37.6	
4	40	20.1	26	26.5	14	13.9	
≥5	38	19.1	20	20.4	18	17.8	

* *p* < 0.05.

**Table 4:** Self-directed learning and problem-solving abilities between the two groups at baseline, pre- and post-intervention through small group work.

	Overall		Control		Intervention	
	Mean	SD	Mean	SD	Mean	SD
Self-directed learning ability						
SM						
Baseline	45.62	7.15	44.74	7.25	46.47	7.00
8-week	46.88	7.62	44.13	7.57	49.55	6.76
16-week	51.30	6.60	52.00	7.51	50.62	6.34
28-week	51.95	7.09	52.21	7.80	51.70	6.35
SC						
Baseline	58.27	7.93	57.84	7.82	58.68	8.07
8-week	59.52	8.22	56.21	7.55	62.73	7.58
16-week	63.31	7.28	63.99	7.81	62.64	6.70
28-week	64.31	7.36	64.13	8.42	64.48	6.19
DL						
Baseline	47.56	8.80	48.04	10.96	47.10	6.03
8-week	48.44	6.25	45.95	6.03	50.86	5.50
16-week	50.01	5.78	51.86	6.04	50.18	5.42
28-week	51.50	5.61	51.38	6.00	51.61	5.23
Overall SDLR						
Baseline	151.45	19.89	150.62	20.73	152.25	19.10
8-week	154.85	20.29	146.30	19.06	163.15	17.94
16-week	165.61	18.58	167.85	19.99	163.45	16.91
28-week	167.76	18.61	167.72	21.12	167.79	15.92
Problem-solving ability						
AAS						
Baseline	39.64	6.45	39.32	6.20	39.94	6.69
8-week	39.44	6.89	42.28	5.81	36.69	6.77
16-week	35.92	6.57	36.63	5.89	35.24	7.13
28-week	33.56	7.96	33.81	8.60	33.33	7.32
PSC						
Baseline	25.63	5.10	25.60	5.41	25.65	4.80
8-week	25.25	5.45	27.31	5.26	23.25	4.89
16-week	22.48	4.79	22.68	4.91	22.29	4.68
28-week	21.19	5.75	21.37	6.29	21.01	5.20
PC						
Baseline	12.67	2.84	12.44	2.67	12.90	3.00
8-week	12.80	3.30	13.39	2.96	12.23	3.53
16-week	12.28	3.35	12.79	3.31	11.78	3.32
28-week	11.34	4.01	11.33	4.07	11.36	3.98
Overall PS						
Baseline	77.94	12.30	77.37	11.95	78.50	12.67
8-week	77.49	13.28	82.97	11.50	72.17	12.77
16-week	70.68	12.27	72.10	11.35	69.31	13.02
28-week	66.09	15.50	66.50	16.71	65.69	14.30

SM: self-management, DL: desire for learning, SC: self-control, SDL: self-directed learning, AAS: approach-avoidance style, PSC: problem-solving confidence, PC: personal control, PS: problem-solving, SDLR: self-directed learning readiness.

**Table 5:** Changes of self-directed learning and problem-solving abilities through small group work between two groups at four study periods (*n* = 199).

Independent Samples *t*-Test				Repeated Measures		
Periods	*t*	*p*	95% CI	Periods	Mauchly’s Test	Tests of Within-Subject Effects
SM				SM		
Baseline	1.70	0.090	−0.27 to 3.71	Baseline to 8th week		F(1,197) = 15.87, *p* < 0.001 ***
8th week	5.35	<0.001 ***	3.42 to 7.42	8th week to 16th week		F(1,197) = 67.65, *p* < 0.001 ***
16th week	−1.40	0.164	−3.32 to 0.57	16th week to 28th week		F(1,197) = 1.21, *p* = 0.273
28th week	−0.51	0.165	−2.50 to 1.48	4 periods	ꭓ2 (5) = 22.54, *p* < 0.001 ***	F(3,591) = 22.14, *p* < 0.001 ***
SC				SC		
Baseline	0.75	0.453	−1.38 to 3.07	Baseline to 8th week		F(1,197) = 27.53, *p* < 0.001 ***
8th week	6.08	<0.001 ***	4.40 to 8.63	8th week to 16th week		F(1,197) = 70.89, *p* < 0.001 ***
16th week	−1.30	0.194	−3.38 to 0.69	16th week to 28th week		F(1,197) = 3.51, *p* = 0.063
28th week	0.33	0.745	−1.73 to 2.42	4 periods	ꭓ2 (5) = 36.94, *p* < 0.001 ***	F(3,591) = 20.45, *p* < 0.001 ***
DL				DL		
Baseline	−0.75	0.456	−3.40 to 1.52	Baseline to 8th week		F(1,197) = 20.09, *p* < 0.001 ***
8th week	6.00	<0.001 ***	3.30 to 6.52	8th week to 16th week		F(1,197) = 99.65, *p* < 0.001 ***
16th week	−2.06	0.041 *	−3.28 to −0.08	16th week to 28th week		F(1,197) = 9.08, *p* = 0.003 *
28th week	0.30	0.768	−1.34 to 1.81	4 periods	ꭓ2 (5) = 192.40, *p* < 0.001 ***	F(3,591) = 15.69, *p* < 0.001 ***
Overall SDL				Overall SDL		
Baseline	0.65	0.519	−3.95 to 7.20	Baseline to 8th week		F(1,197) = 31.93, *p* < 0.001 ***
8th week	−6.42	<0.001 ***	11.68 to 22.03	8th week to 16th week		F(1,197) = 100.49, *p* < 0.001 ***
16th week	−1.62	0.108	−9.57 to 0.77	16th week to 28th week		F(1,197) = 4.87, *p* = 0.028 *
28th week	0.03	0.980	−5.15 to 5.29	4 periods	ꭓ2 (5) = 53.03, *p* < 0.001 ***	F(3,591) = 26.01, *p* < 0.001 ***
AAS				AAS		
Baseline	0.67	0.503	−1.19 to 2.42	Baseline to 8th week		F(1,197) = 35.96, *p* < 0.001 ***
8th week	−6.25	<0.001 ***	−7.34 to −3.82	8th week to 16th week		F(1,197) = 24.31, *p* < 0.001 ***
16th week	−1.51	0.134	−3.23 to 0.44	16th week to 28th week		F(1,197) = 0.69, *p* = 0.406
28th week	−0.42	0.673	−2.72 to 1.76	4 periods	ꭓ2 (5) = 41.26, *p* < 0.001 ***	F(3,591) = 12.01, *p* < 0.001 ***
PSC				PSC		
Baseline	0.71	0.944	−1.38 to 1.48	Baseline to 8th week		F(1,197) = 26.15, *p* < 0.001 ***
8th week	−5.64	<0.001 ***	−5.48 to −2.64	8th week to 16th week		F(1,197) = 33.93, *p* < 0.001 ***
16th week	−0.58	0.561	−1.74 to 0.94	16th week to 28th week		F(1,197) = 0.002, *p* = 0.960
28th week	−0.44	0.663	−1.97 to 1.26	4 periods	ꭓ2 (5) = 29.16, *p* < 0.001 ***	F(3,591) = 11.76, *p* < 0.001 ***
PC				PC		
Baseline	1.15	0.252	−0.33 to 1.26	Baseline to 8th week		F(1,197) = 12.30, *p* = 0.001 ***
8th week	−2.51	0.013 *	−2.07 to −0.25	8th week to 16th week		F(1,197) = 0.136, *p* = 0.713
16th week	−2.14	0.034 *	−1.93 to −0.08	16th week to 28th week		F(1,197) = 4.21, *p* = 0.042 *
28th week	0.05	0.958	−1.10 to 1.16	4 periods	ꭓ2 (5) = 34.01, *p* < 0.001 ***	F(3,591) = 4.76, *p* = 0.003 *
Overall PS				Overall PS		
Baseline	−0.65	0.519	−2.32 to 4.57	Baseline to 8th week		F(1,197) = 37.84, *p* < 0.001 ***
8th week	6.28	<0.001 ***	−14.20 to −7.40	8th week to 16th week		F(1,197) = 26.34, *p* < 0.001 ***
16th week	1.62	0.108	−6.21 to 0.62	16th week to 28th week		F(1,197) = 0.89, *p* = 0.346
28th week	−0.37	0.715	−2.16 to 3.55	4 periods	ꭓ2 (5) = 45.11, *p* < 0.001 ***	F(3,591) = 12.73, *p* < 0.001 ***

SM: self-management, DL: desire for learning, SC: self-control, SDL: self-directed learning, AAS: approach-avoidance style, PSC: problem-solving confidence, PC: personal control, PS: problem-solving, SDLR: self-directed learning readiness. * *p* < 0.05; *** *p* < 0.001.

## Data Availability

The data presented in this study are available on request from the corresponding author. The data are not publicly available due to privacy reason.

## Supplementary Materials

The following supporting information can be downloaded at https://www.mdpi.com/article/10.3390/ijerph19020720/s1. Supplementary Materials S1: An example of case scenario using PBL approach.
