# Supplementary material for: Online Problem-Based Learning Intervention on Self-Directed Learning and Problem-Solving through Group Work: A Waitlist Controlled Trial
Source: Int J Environ Res Public Health. 2022 Jan 10;19(2):720. doi: 10.3390/ijerph19020720 (PMC8775567; doi:10.3390/ijerph19020720)
Supplement: Supplementary file 1 [file ijerph-19-00720-s001.zip › ijerph-1474621-supplementary.pdf]

Supplementary Materials S1

Before a semester starts, the course coordinator will prepare adequate learning materials and guidelines for students. In this study, there are six case scenarios using problem-based learning to enhance students' learning. The sample case scenario was illustrated below.

Sample case scenario

Lesson 1

Expected learning objectives: (tutor can allow students or guide students to set their own achievable objectives)

Students are able to

- Understand their roles and responsibility in a group project
- Understand that they will have oral presentation about the case and the topic at the end of the course
- Distribute tasks to student groups and establish objectives for next meeting

The sample case scenario related to obesity for Lesson 2:

Ted is a 12-year-old boy. He visits your primary clinic with his mother today. Ted is 180 pounds and 140 cm tall. Ted cried that he has no friends in school.

Preparation for Lesson 2

Expected learning objectives:

Students are able to

- understand the common health concerns in a 12-year-old teen.
- identify possible health problem(s) attributed to obesity.
- perform relevant physical assessment for obesity

The sample case scenario for Lesson 3:

Ted appears very upset due to his appearance. He feels that he is being bullied. He cried that he has no friends in school.

Preparation for Lesson 3

Expected learning objectives:

Students are able to

- discuss physical impacts due to obesity and related physical health needs
- discuss psychosocial impacts due to obesity and related psychosocial health needs
- discuss the consequence if the health problem is not resolved.

The sample case scenario for Lesson 4:

Ted likes eating snacks and cake. He understands that he needs to control his weight. You are his nurse and are going to discuss with him about weight control programme.

Preparation for Lesson 4

Expected learning objectives:

Students are able to

- develop an interventional plan for appropriate care and treatment
- discuss effective strategies to reduce the vulnerabilities
- apply available resources from community

The sample case scenario for Lesson 5:

Ted works out the plan for his weight control. He comes back for the follow up and further evaluation.

Preparation for Lesson 5

Expected learning objectives:

- understand the difficulties in working out the plan and the coping strategies used by Ted
- Give positive support and appreciation if appropriate
- Evaluate if the interventional plan is workable

Preparation for Lesson 6

Expected learning objectives:

- Students are able to gather and conclude all information they have learned.

Lesson 6: Reflection

Students are required to reflect their learning through individual and group learning throughout the semester. They will have feedback from their tutor.
